# Blood volume expansion does not explain the increase in peak oxygen uptake induced by 10 weeks of endurance training

**DOI:** 10.1007/s00421-020-04336-2

**Published:** 2020-03-14

**Authors:** Øyvind Skattebo, Anders Wold Bjerring, Marius Auensen, Sebastian Imre Sarvari, Kristoffer Toldnes Cumming, Carlo Capelli, Jostein Hallén

**Affiliations:** 1 Department of Physical Performance, Norwegian School of Sport Sciences, Post box 4014 Ullevål Stadion, 0806 Oslo, Norway; 2 Center for Cardiological Innovation, Oslo University Hospital, Oslo, Norway; 3 University of Oslo, Oslo, Norway

**Keywords:** Blood volume, Cardiac output, Echocardiography, Haemoglobin mass, Maximal oxygen uptake, Peripheral adaptations, Supine cycling

## Abstract

**Purpose:**

The endurance training (ET)-induced increases in peak oxygen uptake ($\dot{V}$O_2peak_) and cardiac output ($\dot{Q}$_peak_) during upright cycling are reversed to pre-ET levels after removing the training-induced increase in blood volume (BV). We hypothesised that ET-induced improvements in $\dot{V}$O_2peak_ and $\dot{Q}$_peak_ are preserved following phlebotomy of the BV gained with ET during supine but not during upright cycling. Arteriovenous O_2_ difference (a-$\bar{\text{v}}$O_2_diff; $\dot{V}$O_2_/$\dot{Q}$), cardiac dimensions and muscle morphology were studied to assess their role for the $\dot{V}$O_2peak_ improvement.

**Methods:**

Twelve untrained subjects ($\dot{V}$O_2peak_: 44 ± 6 ml kg^−1^ min^−1^) completed 10 weeks of supervised ET (3 sessions/week). Echocardiography, muscle biopsies, haemoglobin mass (Hb_mass_) and BV were assessed pre- and post-ET. $\dot{V}$O_2peak_ and $\dot{Q}$_peak_ during upright and supine cycling were measured pre-ET, post-ET and immediately after Hb_mass_ was reversed to the individual pre-ET level by phlebotomy.

**Results:**

ET increased the Hb_mass_ (3.3 ± 2.9%; *P* = 0.005), BV (3.7 ± 5.6%; *P* = 0.044) and $\dot{V}$O_2peak_ during upright and supine cycling (11 ± 6% and 10 ± 8%, respectively; *P* ≤ 0.003). After phlebotomy, improvements in $\dot{V}$O_2peak_ compared with pre-ET were preserved in both postures (11 ± 4% and 11 ± 9%; *P* ≤ 0.005), as was $\dot{Q}$_peak_ (9 ± 14% and 9 ± 10%; *P* ≤ 0.081). The increased $\dot{Q}$_peak_ and a-$\bar{\text{v}}$O_2_diff accounted for 70% and 30% of the $\dot{V}$O_2peak_ improvements, respectively. Markers of mitochondrial density (CS and COX-IV; *P* ≤ 0.007) and left ventricular mass (*P* = 0.027) increased.

**Conclusion:**

The ET-induced increase in $\dot{V}$O_2peak_ was preserved despite removing the increases in Hb_mass_ and BV by phlebotomy, independent of posture. $\dot{V}$O_2peak_ increased primarily through elevated $\dot{Q}$_peak_ but also through a widened a-$\bar{\text{v}}$O_2_diff, potentially mediated by cardiac remodelling and mitochondrial biogenesis.

## Introduction

During whole-body exercise, the oxidative capacity of skeletal muscle exceeds the oxygen (O_2_) delivery, as illustrated by the two-fold higher mass-specific O_2_ delivery and peak O_2_ uptake ($\dot{V}$O_2peak_) during dynamic one-legged knee-extension compared to cycling exercise (approximately 2.5 vs 20 kg active muscle mass, respectively) (Boushel and Saltin 2013; Cardinale et al. 2019). Yet, endurance training (ET) induces remarkable increases in mitochondrial enzymes and capillary density, commonly improving these by ~ 40% and ~ 10–20% after a few months of ET, respectively (Granata et al. 2018; Klausen et al. 1981). Such adaptations are likely more important for endurance performance than $\dot{V}$O_2peak_, but despite a long-standing debate (Bassett and Howley 2000), it remains uncertain whether $\dot{V}$O_2peak_ is limited by central or by combined central–peripheral factors. According to the Fick equation, every change in $\dot{V}$O_2peak_ is matched by a concomitant change in peak cardiac output ($\dot{Q}$_peak_) and/or arteriovenous O_2_ difference (a-$\bar{\text{v}}$O_2_diff). Most studies find $\dot{Q}$_peak_ increased following short-term ET, whereas more heterogeneous findings exist for the a-$\bar{\text{v}}$O_2_diff (Montero and Diaz-Canestro 2016).

At the commencement of ET, improvements in stroke volume (SV) and $\dot{Q}$_peak_ are thought to be mainly facilitated by the blood volume (BV) expansion that increases venous return and preload to the heart and its filling rates (Bonne et al. 2014; Krip et al. 1997). However, cardiac remodelling has been reported already after 3 months of ET, which, together with the reduced pericardial constraints and enhanced cardiac compliance after long periods of ET (Arbab-Zadeh et al. 2014), suggests multifactorial mechanisms for the enhancement of $\dot{Q}$_peak_ with ET (Saltin et al. 1968).

The ET-induced increase in BV can be removed by phlebotomy to assess its importance for the ET-induced changes in $\dot{Q}$_peak_ and $\dot{V}$O_2peak_. When using this experimental design, the 7–10% improvement in $\dot{V}$O_2peak_ and $\dot{Q}$_peak_ after 6 weeks of ET were reversed to pre-ET levels after phlebotomy (Bonne et al. 2014; Montero et al. 2015a). Concomitantly, no change in cardiac morphology (Bonne et al. 2014) nor in the a-$\bar{\text{v}}$O_2_diff were found despite robust peripheral adaptations (Montero et al. 2015a). Therefore, the authors suggested that the improvement in $\dot{V}$O_2peak_ was explained by increased $\dot{Q}$_peak_ attributed to the BV expansion alone. However, acute hypovolemia due to phlebotomy may increase the sympathetic tone leading to arteriolar and venous constriction (Fortrat et al. 1998; Zollei et al. 2004) and offset an ET-induced drop in peripheral vascular resistance in these studies. In this context, reduced BV and impaired haemodynamic control after bed rest can lead to a reduction in $\dot{Q}$_peak_ and $\dot{V}$O_2peak_ during upright cycling (Saltin et al. 1968), which is reversed when measured during supine cycling (Bringard et al. 2010) due to gravitational effects on central BV, preload and thus SV (Warburton et al. 2002). The supine position increases the central venous pressure and the baroreflex loading compared to the upright position (Ray et al. 1993), thus potentially avoiding increases in sympathetic tone and total peripheral resistance in the face of reduced BV.

We investigated the importance of the ET-induced increases in haemoglobin mass (Hb_mass_) and BV for the changes in $\dot{V}$O_2peak_ and $\dot{Q}$_peak_. Maximal exercise was conducted before and after removing an amount of blood corresponding to the measured individual increase in Hb_mass_ induced by ET. We hypothesised that the increases in $\dot{V}$O_2peak_ and $\dot{Q}$_peak_ would return to pre-ET levels after phlebotomy during upright cycling but remain elevated during supine cycling owing to improved venous return. Furthermore, we hypothesised that the change in $\dot{V}$O_2peak_ is mostly facilitated by elevated $\dot{Q}$_peak_, but also by a widened a-$\bar{\text{v}}$O_2_diff. Potential mechanisms for the changes in a-$\bar{\text{v}}$O_2_diff and $\dot{Q}$_peak_ were studied in muscle biopsies (capillarisation and mitochondrial enzymes) and by echocardiography, respectively.

## Materials and methods

### Ethical approval

The study was approved by the Ethics Committee of the Norwegian School of Sport Sciences (ref. 13-220817) and The Norwegian Centre for Research Data (ref. 55151). Oral and written informed consents were obtained from all subjects before the start of this investigation, which was carried out in accordance with the Declaration of Helsinki.

### Subjects

Twelve untrained subjects, defined as conducting ≤ 1 ET session per week during the previous year, were recruited and completed the ET period (7♂; age: 29.2 ± 5.9 years; weight: 72.4 ± 13.1 kg; height: 1.75 ± 0.11 m; body fat: 27 ± 5%; $\dot{V}$O_2peak_: 44.2 ± 5.9 ml kg^−1^ min^−1^). One subject performed all tests except the phlebotomy procedure and the post-phlebotomy testing. Therefore, data from this individual were used only when presenting individual responses and the muscle biopsy analyses. All subjects were non-smokers and reported no contraindications to ET or maximal exercise testing.

### Experimental design

**Fig. 1:**
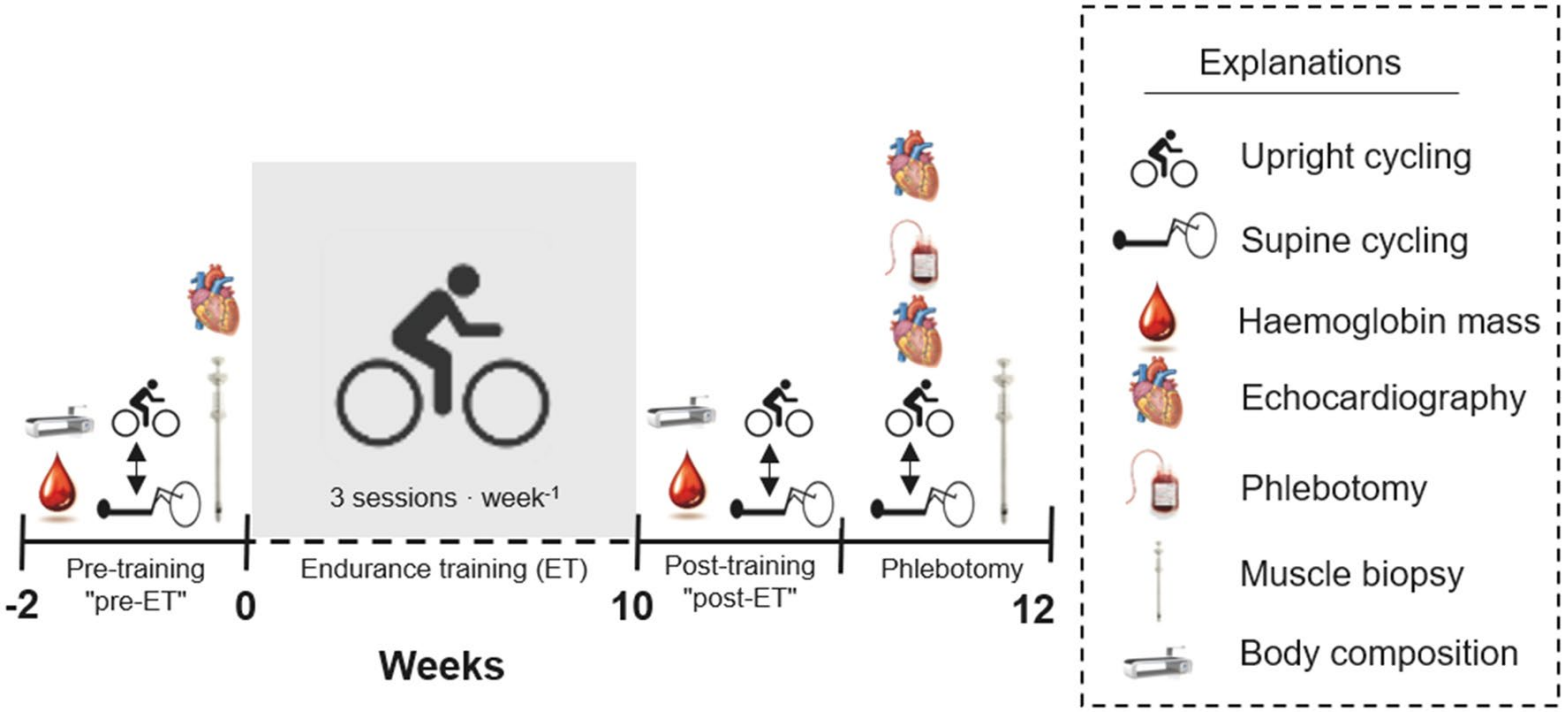
The experimental design of the study. During the phlebotomy trial, echocardiography was conducted first (the post-ET echocardiography), following which the subjects were phlebotomised. This was followed by a second echocardiography. The first of two cycling exercises was initiated precisely 45 min after phlebotomy

The experimental design is summarised in Fig. 1. Before and after 10 weeks of ET, Hb_mass_, BV, body composition and cardiac dimensions were assessed, and a biopsy from the *m. vastus lateralis* was obtained under resting conditions. Maximal exercise testing was performed during upright and supine cycling before and after ET as well as directly after removing the BV necessary to counteract the individual increase in Hb_mass_ elicited by the ET. Before pre-ET measurements, all subjects were familiarised with supine cycling (two sessions) and maximal exercise during upright cycling.

### Exercise training

**Fig. 2:**
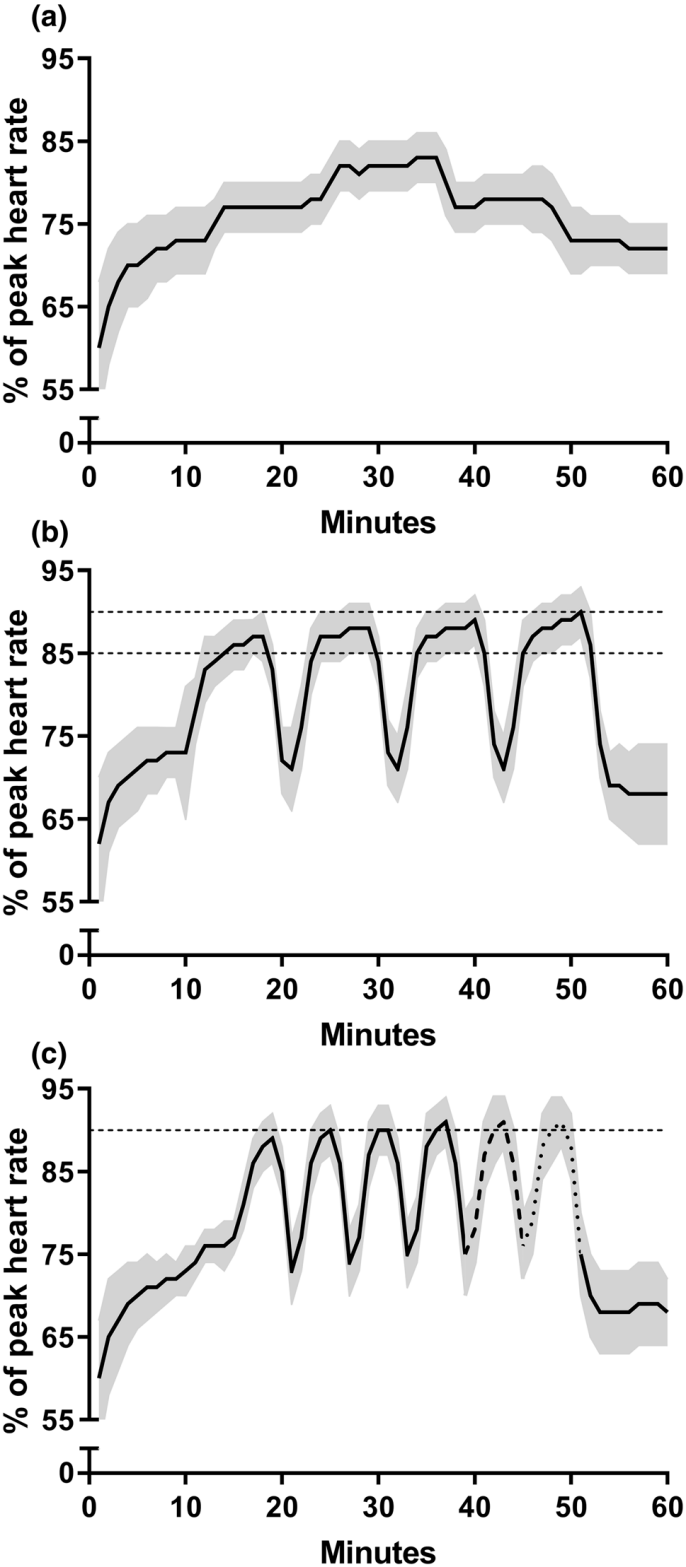
Percentage of peak heart rate during continuous moderate intensity (**a**); 8-min interval (**b**); and 4-min interval (**c**) sessions conducted during the training period. The black lines and the grey-shaded areas denote their mean values and standard deviations, respectively

During the 10-week ET period, the subjects underwent three supervised training sessions per week. Session 1 consisted of 60 min of continuous exercise between 70–80% of peak heart rate (HR_peak_) (Fig. 2a). Session 2 included 4 × 8 min intervals with a target intensity between 85–90% of HR_peak_ (Fig. 2b). Lastly, session 3 consisted of 4–6 × 4 min intervals with a target intensity ≥ 90% of HR_peak_ (four repetitions in weeks 1–3, 5 repetitions in weeks 4–6, and six repetitions in weeks 7–9; Fig. 2c). The intervals were interspersed by 3 or 2 min active recovery at ~ 70% of HR_peak_, respectively. A short tapering was performed in week 10 to maximise performance during the post-ET testing. The continuous session was shortened to 40 min, and the number of repetitions during the 8 and 4 min intervals were reduced to three and four repetitions, respectively. All subjects conducted 27–30 ET sessions (compliance: 94.4 ± 3.6%).

### Measurements and procedures

#### Body composition

Body composition was assessed by dual-energy X-ray absorptiometry (Lunar iDXA, enCORE software version 17; GE Healthcare, Chicago, IL, USA) after overnight fasting.

#### Haematology and blood withdrawal

Blood from an antecubital vein was collected in the morning under standardised, seated conditions. Haematological variables were analysed for EDTA blood (3 ml; BD, Franklin Lakes, NJ, USA) using a Sysmex XN-9000 (Sysmex, Kobe, Japan). Serum samples (SST II Advance 5 ml; BD) were placed at room temperature for coagulation (30 min), centrifuged at 1500 G for 10 min and stored at 4 °C until analysed for ferritin concentration (Advia Chemistry XPT; Siemens Healthineers, Erlangen, Germany).

Hb_mass_ was measured in duplicate on separate days using a carbon monoxide (CO) rebreathing method (Prommer and Schmidt 2007; Schmidt and Prommer 2005). First, the subjects rested seated for 10 min, followed by capillary blood sampling in two 125-μl pre-heparinised tubes (Clinitubes; Radiometer, Copenhagen, Denmark) from a pre-heated fingertip. The subjects then inhaled a bolus of 1.0 (♀) or 1.2 (♂) ml per kg body weight of 99.97% chemically pure CO (AGA Norge, Oslo, Norway) administered via a 100-ml plastic syringe (Omnifix; Braun, Kronberg im Taunus, Germany) to a spirometer (Blood tec GmbH, Germany). In this closed circuit, the CO was rebreathed for 2 min together with 3 l of pure O_2_ (AGA Norge) while checking for leakages using a CO analyser (Draeger, Lübeck, Germany). Two capillary blood samples were collected, 6 and 8 min after the administration of CO. All blood samples were immediately analysed in duplicate for percent carboxyhaemoglobin using an ABL80 CO-OX FLEX (Radiometer). After rebreathing, the CO not absorbed by the body was calculated by multiplying the CO concentration of the rebreathing bag by the bag volume and the subject’s estimated residual lung volume (Miller et al. 1998). The CO exhaled between the time point of disconnecting from the spirometer to the blood sampling was estimated by multiplying the difference in end-tidal CO concentration before and after rebreathing by the estimated alveolar ventilation (West 2008). The Hb_mass_ was calculated by dilution of CO in blood (Schmidt and Prommer 2005) with correction for loss of CO to myoglobin (0.3% of the administered CO per minute) (Prommer and Schmidt 2007). The coefficient of variation of the duplicate Hb_mass_ determinations, expressed as the percent typical error (standard deviation of the difference scores/$\sqrt{2}$), was 1.10%. To derive intravascular volumes (BV; red blood cell volume, RBCV; plasma volume, PV), the formulae given by Siebenmann et al. (1985) were used with no correction for venous to whole-body haematocrit.

The phlebotomy trial was conducted 3.9 ± 2.2 days after the post-ET trial. A BV equal to each individual’s ET-induced increase in Hb_mass_ (ΔHb_mass_/[Hb]) was removed by phlebotomy via an 18 G catheter (BD) indwelling in an antecubital vein. Immediately after the phlebotomy, echocardiography was conducted, followed by exercise testing starting 45 min after the phlebotomy.

#### Exercise testing

Upright and supine cycling were conducted in random counterbalanced order on the same day, interspersed by 45 min of passive rest. During upright cycling (Excalibur Sport; Lode B.V., Groningen, The Netherlands), the test started with a 3-min resting measurement while seated on the bike. Thereafter, three 5-min submaximal workloads (♂ 50–150 W, ♀ 50–100 W) were conducted directly followed by a maximal test with step-increments of 25 W every minute until exhaustion. The mean workload during the last 60 s was defined as the peak power output ($\dot{W}$_peak_). During supine cycling (Angio 2000; Lode B.V.), the subjects were fastened with a four-point harness lying on a bench. The axis of rotation and thus the legs were raised ~ 20 cm above the heart. The structure of the protocol was similar to upright cycling, with the submaximal workloads (♂ 50–100 W, ♀ 50–75 W) followed by step-increments of 20 W every minute. After reaching exhaustion, the subjects cycled at 40–50 W for 5 min to speed up their recovery. $\dot{V}$O_2_ was measured over the last 2.5 min at each submaximal stage and continuously during the resting measurements and incremental tests, using open-circuit indirect calorimetry with a mixing chamber (Oxycon Pro; Jaeger Instrument, Friedberg, Germany) (Foss and Hallén 2005). Before each test, the gas analysers and flow transducer were calibrated according to the instruction manual.

SV, HR and $\dot{Q}$ were continuously monitored by impedance cardiography and an integrated electrocardiogram using a PhysioFlow Q-link device (Manatec Biomedical, Paris, France). This method is evaluated against the direct Fick method (Charloux et al. 2000; Richard et al. 2001; Siebenmann et al. 2015) and uses the cyclic variations in transthoracic impedance during the cardiac cycle to estimate SV, as these pulsatile variations represent the changes in volume and velocity of the aortic BV (Charloux et al. 2000; Richard et al. 2001). Six electrodes (PF-50; Manatec Biomedical) were placed on each subject’s neck, chest and back after the skin was cleaned with alcohol and rubbed with an abrasive ECG preparation gel (Custo prep; Custo med, Ottobrunn, Germany). Equal placement of electrodes in all tests was ensured by tracking their positions on transparent plastic sheets according to skin and anatomical landmarks. The subjects wore a tight mesh t-shirt to avoid displacement of the electrodes and their attached leads. A fan was placed in front of the subjects for heat dissipation to counteract the accumulation of sweat during exercise, to ensure that the electrodes maintained adhesiveness throughout the test. After instrumentation, the subjects rested on the ergometer for 5 min before autocalibration of the software (version 2.7.4). Immediately after the autocalibration, blood pressure was measured in duplicate (ProBP 3400 series; Welch Allyn, Skaneateles, NY, USA) and the mean values were fed to the software.

$\dot{V}$O_2_, SV, HR and $\dot{Q}$ were recorded using 10-s averages. On the submaximal workloads, the average of the last 2 min served as the steady-state values. During the incremental tests to exhaustion, the highest 30-s average was taken as the peak value. The a-$\bar{\text{v}}$O_2_diff was calculated as the ratio between $\dot{V}$O_2_ and $\dot{Q}$ according to the Fick equation. The peak capillary blood lactate concentration ([La]_peak_) was measured 1 min after exhaustion (Biosen C-line; EKF Diagnostic, Cardiff, UK). The typical error for $\dot{W}$_peak_ and $\dot{V}$O_2peak_ measured during upright cycling (familiarisation vs pre-ET) was 3.0% and 2.9%, respectively.

#### Transthoracic echocardiography

All subjects underwent three echocardiographic studies (pre-ET, post-ET and directly after phlebotomy) (Vivid E95; GE Vingmed Ultrasound AS, Horten, Norway) using a 2.5 MHz (M5Sc) and an active matrix 4D volume-phased array transducer. Echocardiographic views were obtained using greyscale harmonic imaging according to the recommendations of the European Association of Cardiovascular Imaging (Lang et al. 2015). Recordings were digitally stored for offline post hoc analysis (EchoPac; GE Vingmed Ultrasound AS) carried out by a blinded observer. From 2D echocardiography, left ventricular (LV) dimensions and LV diastolic function parameters were assessed. Right ventricular (RV) areas and fractional area change were assessed in the four-chamber view. Tissue Doppler was used to assess wall motion velocities at the mitral annulus level and mean values from the septal and lateral walls are reported. LV mass was calculated using Devereux’ formula (Devereux et al. 1986). 3D data sets, including LV volumes and ejection fraction, were obtained using a dedicated semi-automated algorithm.

Strain analysis was performed using 2D speckle-tracking echocardiography by automatic tracking of acoustic markers on a frame-by-frame basis throughout the cardiac cycle. The endocardial borders were traced in the end-systolic frame of the 2D images from the apical 4-, 2-chamber, and apical long-axis views for the assessment of longitudinal strain. The operator manually adjusted segments where the automatic tracking failed. Peak systolic LV global longitudinal strain was averaged from 16 LV segments. The frame rate was 61 ± 5 Hz.

#### Skeletal muscle biopsy

Biopsies (~ 100–200 mg) were collected from the mid-portion of *m. vastus lateralis* after local anaesthesia, using the Bergström technique with manual suction (*n* = 10). The tissue was immediately dissected free from visible fat and connective tissue. An appropriate sample for immunohistochemistry was embedded in OCT (CellPath, Newtown, UK) and quickly frozen in isopentane cooled on liquid nitrogen to freezing point (approx. − 120 °C). Tissue allocated for Western Blotting was immediately snap-frozen in liquid nitrogen. All tissue samples were stored at  −  80 °C until further analyses.

#### Immunohistochemistry

Serial 8 μm transverse cross-sections were cut at − 20 °C (Leica CM1860 UV; Leica Biosystems, Danvers, MA, USA), mounted on microscope slides (Superfrost Plus; Thermo Fischer Scientific, Waltham, MA, USA), air-dried and stored at − 80 °C until analyses. The sections were blocked for 60 min with 1% bovine serum albumin (Sigma Life Science, St Louis, MO, USA) in a phosphate-buffered saline (Sigma Life Science) and 0.05% Tween-20 (VWR, West Chester, PA, USA) solution (PBS-t). Primary antibodies against (1) myosin heavy chain type 1 (1:500 dilution; BA-D5, obtained from DSHB, Iowa City, IA, USA) (Schiaffino et al. 1989) and dystrophin (1:500; ab15277; Abcam, Cambridge, UK) or (2) the endothelial marker CD31 (1:100; M0823; Dako A/S, Glostrup, Denmark) and dystrophin were diluted in the blocking solution and incubated overnight at 4 °C. The sections were then washed 3 × 10 min in PBS-t, incubated with secondary antibodies (1:200; Alexa Fluor 488, A11001 and A11012; Invitrogen Molecular Probes, Carlsbad, CA, USA) for 60 min and again washed 3 × 10 min in PBS-t before mounted with Prolong Gold antifade reagent with DAPI (Life Technologies Corp., Carlsbad, CA, USA) and covered with glass. The sections were visualised under a 10 × /0.30 NA air objective (UplanFL N; Olympus corp.; Tokyo, Japan) and micrographed using a high-resolution digital camera (DP72; Olympus corp.) attached to a microscope (BX61; Olympus corp.) with a fluorescence light source (X-Cite 120 PC Q; EXFO Photonic Solution Inc., ON, Mississauga, Canada). Fibre cross-sectional areas, fibre types and manual identification of capillaries were conducted and analysed using TEMA software (CheckVision, Denmark). The investigator was blinded for subject identity and time point. Capillarisation was expressed as capillary-to-fibre ratio, capillaries in contact with each fibre and capillary density (capillaries per mm^2^). A mean of 198 ± 56 (range 96–345) fibres were analysed for each cross-section.

#### Protein immunoblot

For Western blotting analyses, ~ 60 mg of muscle tissue was homogenised in 1 ml T-PER (Tissue Protein Extraction Reagent, 78510; Thermo Fischer Scientific) and 20 μL Halt Protease & Phosphatase Inhibitor Cocktail (78440; Thermo Fischer Scientific). The tissue lysate was extracted, aliquoted and stored at − 80 °C until further analyses. The protein concentration was measured using a commercial kit (Bio-Rad DC Protein Assay, 5000116; Bio-Rad Laboratories, Hercules, CA, USA) and a FLUOstar Omega microplate reader (BMG Labtech, Ortenberg, Germany). Standard Western blotting procedures were applied for quantification of citrate synthase (CS), cytochrome c oxidase subunit 4 (COX-IV) and hydroxyacyl-CoA dehydrogenase (HAD): 20 μg of protein was separated by 4–12% gradient Bis–Tris gels (Invitrogen, Life Technologies) for ~ 45 min at 200 V in cold buffer (NuPage MES SDS Running Buffer; Invitrogen, Life Technologies). Proteins were subsequently transferred onto a PVDF membrane (Bio-Rad Laboratories) at 30 V for 90 min in cold buffer (NuPage Transfer Buffer; Invitrogen, Life Technologies). Membranes were blocked at room temperature for 2 h in a 5% fat-free skimmed milk (Merck, Darmstadt, Germany) and 0.1% TBS-t solution (TBS: Bio-Rad Laboratories; Tween-20: VWR). Thereafter, the membranes were divided into three pieces based on molecular weight (Protein Ladder 310005; GeneON, Ludwigshafen am Rhein, Germany) and then incubated overnight (4 °C) with primary antibodies against CS (1:4000; ab96600; Abcam), COX-IV (1:2000; ab16056; Abcam) or HAD (1:8000; ab154088; Abcam). An anti-rabbit IgG (1:3000; 7074S; Cell Signaling Technology, Danvers, MA, USA) secondary antibody was applied for 1 h at room temperature followed by visualisation using an HRP detection system (Super Signal West Dura Extended Duration Substrate; Thermo Fischer Scientific). All antibodies were diluted in a 1% fat-free skimmed milk and 0.1% TBS-t solution. Between steps, membranes were washed in 0.1% TBS-t and TBS solutions. Chemiluminescence was detected using the ChemiDoc MP system with band-intensities quantified using Image Lab 5.1 software (Bio-Rad Laboratories). Pre- and post-samples were loaded on the same gel in duplicate, and mean values were used for statistical analysis.

#### Statistical analyses

Data in text and tables are presented as mean ± standard deviation (SD) and in graphs as mean ± standard error of the mean. The data were initially assessed for normal distribution using the D'Agostino-Pearson test. For variables only measured pre-ET and post-ET, group changes were analysed with a paired Student’s *t* test. Peak and submaximal responses measured on three time points were analysed using repeated measures ANOVA and two-way repeated measures ANOVA (workload x time point), respectively. ANOVAs were followed by the Dunnett’s multiple comparisons test, comparing the control situation (pre-ET) with the post-ET and the phlebotomy trial. The alpha level was set to ≤ 0.05 and values between > 0.05 and ≤ 0.10 were considered to indicate trends. GraphPad Prism 8 (GraphPad Software, CA, USA) was used for statistical analysis.

## Results

### Body composition

Subjects’ weight was reduced by 1.4 ± 1.8 kg (*P* = 0.028) during the ET period, which was entirely accounted for by a reduction in fat mass (− 1.8 ± 1.4 kg; *P* = 0.002; Table 1) as lean body mass remained unchanged (0.1 ± 1.3 kg; *P* = 0.895).

**Table 1:** Haematological variables and body composition measured before and after 10 weeks of endurance training

	Pre-training (mean ± SD)	Post-training (mean ± SD)	% Change (mean ± SD)
Body composition			
Body weight (kg)	74.2 ± 12.1	72.7 ± 11.3	− 1.8 ± 2.6*
Lean mass (kg)	51.2 ± 9.0	51.3 ± 9.5	0.0 ± 2.6
2-Leg lean mass (kg)	18.0 ± 3.9	18.0 ± 4.0	− 0.2 ± 1.8
Fat mass (kg)	20.5 ± 4.3	18.8 ± 4.4	− 9.0 ± 8.2*
Haematology			
Hb_mass_ (g)	795 ± 196	820 ± 196	3.3 ± 2.9*
BV (ml)	5098 ± 929	5279 ± 947	3.7 ± 5.6*
RBCV (ml)	2388 ± 548	2370 ± 528	− 0.3 ± 5.0
PV (ml)	2712 ± 432	2909 ± 464	7.5 ± 9.2*
[Hb] (g dl^−1^)	15.5 ± 1.5	15.4 ± 1.3	− 0.2 ± 3.6
Haematocrit (%)	46.5 ± 3.7	44.6 ± 3.3	− 3.8 ± 4.5*
MCHC (g dl^−1^)	33.3 ± 1.8	34.5 ± 0.9	4.0 ± 4.8*
S-Ferritin (μg l^−1^)	87.7 ± 58.8	86.9 ± 52.9	11.9 ± 41.7

*N* = 11, *BV* blood volume, *[Hb]* haemoglobin concentration, *Hb*_*mass*_ haemoglobin mass, *MCHC* mean corpuscular haemoglobin concentration, *PV* plasma volume, *RBCV* red blood cell volume

*Significant change from pre- to post-training (*P* ≤ 0.05)

### Haematological adaptations

**Fig. 3:**
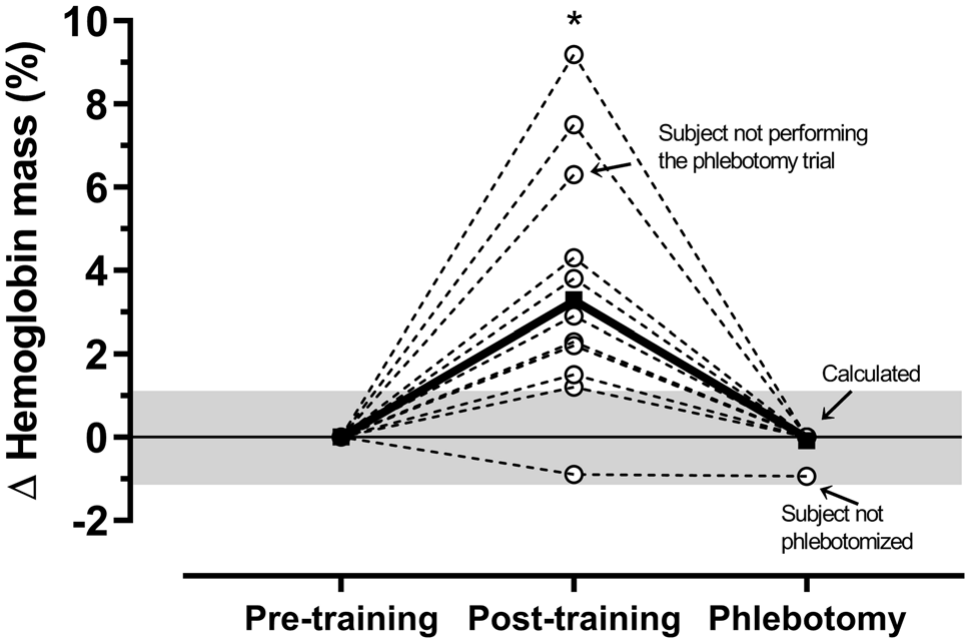
Individual (white circles and dashed lines) and mean changes (black squares and solid line) in haemoglobin mass from before to after 10 weeks of endurance training. The grey area represents the percent typical error for this variable, as calculated from the duplicate measurements (1.10%). *Significantly different from pre-training (*P* = 0.005). *N* = 11–12

The Hb_mass_ increased by 24 ± 22 g (*P* = 0.005; Fig. 3) and BV increased by 181 ± 288 ml (*P* = 0.044; Table 1) from pre- to post-ET. To re-establish pre-ET levels of Hb_mass_ during the phlebotomy exercise trial, 166 ± 139 ml of whole blood was phlebotomised, which caused the BV to be unchanged compared with pre-ET levels (difference: 15 ± 195 ml; *P* = 0.953). Due to a small increase in MCHC (*P* = 0.022) after ET (i.e. higher Hb concentration in the RBCs), the RBCV was decreased (difference: − 89 ± 95 ml; *P* = 0.020) and the PV was slightly increased (difference: 104 ± 192; *P* = 0.174) after phlebotomy compared with pre-ET levels.

### Maximal exercise tests

**Fig. 4:**
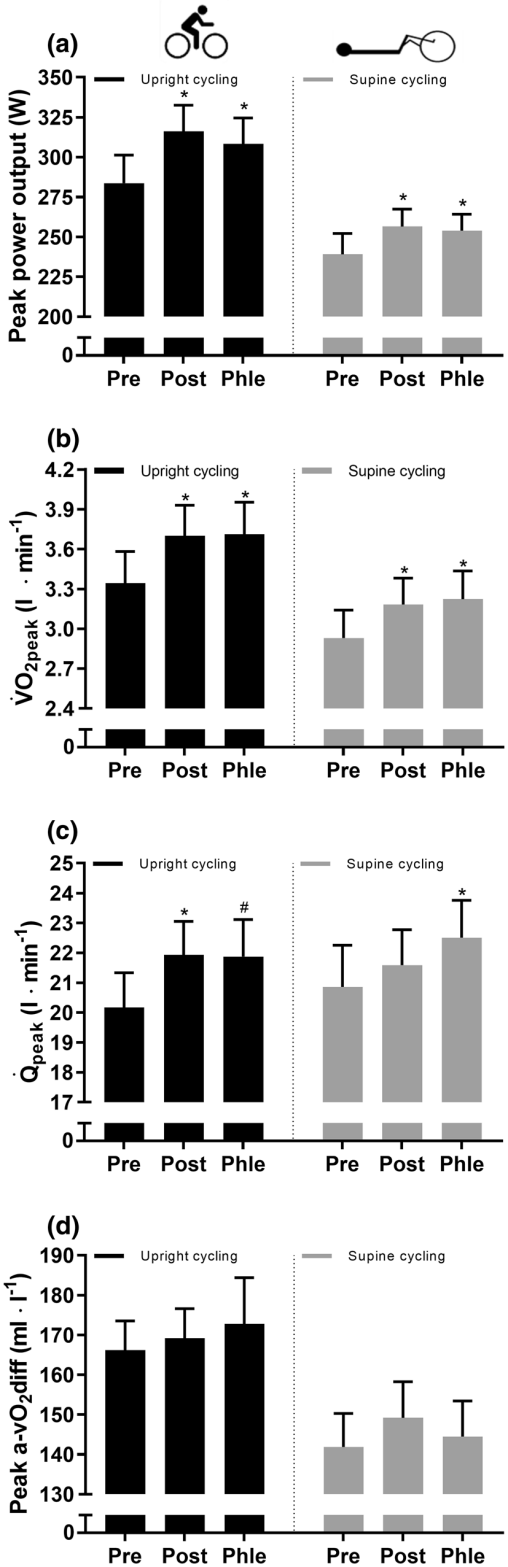
Peak values of power output (**a**); oxygen uptake ($\dot{V}$O_2peak_) (**b**); cardiac output ($\dot{Q}$_peak_) (**c**); and arteriovenous O_2_ difference (a-$\bar{\text{v}}$O_2_diff) (d) during incremental exercise tests to exhaustion before training (Pre), after training (Post) and after training and phlebotomy (Phle). Error bars indicate the standard error of the mean. *Significant change from pre-training (*P* ≤ 0.05). ^#^Trend towards change from pre-training (0.05 < *P* ≤ 0.10). *N* = 11

After ET, $\dot{W}$_peak_ during upright and supine cycling was increased (12 ± 7%; *P* < 0.001 and 8 ± 8%; *P* = 0.014, respectively) and remained elevated after phlebotomy (9 ± 7%; *P* = 0.001 and 7 ± 8%; *P* = 0.048, respectively; Fig. 4a). Similarly, $\dot{V}$O_2peak_ increased by 11 ± 6% (0.36 ± 0.20 l min^−1^; *P* < 0.001) and 10 ± 8% (0.25 ± 0.20 l min^−1^; *P* = 0.003) during upright and supine cycling, respectively, and both remained elevated after phlebotomy (11 ± 4% and 11 ± 9%, respectively; both *P* < 0.005; Fig. 4b). During upright cycling, $\dot{Q}$_peak_ increased by 10 ± 10% (1.8 ± 1.5 l min^−1^; *P* = 0.005) after ET and showed a trend towards remaining elevated after phlebotomy (9 ± 14%; *P* = 0.081; Fig. 4c). During supine cycling, $\dot{Q}$_peak_ was not changed after ET (4 ± 9%; *P* = 0.376) but was increased after phlebotomy compared to pre-ET (9 ± 10%; *P* = 0.023; Fig. 4c). This effect was driven by two outliers lowering the mean value during the post-ET assessment. The peak a-$\bar{\text{v}}$O_2_diff during upright and supine cycling was unchanged after ET, both before (*P* = 0.788 and *P* = 0.228, respectively) and after (*P* = 0.528 and *P* = 0.850, respectively) phlebotomy (Fig. 4d).

At the three measurement time points, a similar level of exertion was evident during the incremental exercise tests, as indicated by similar peak ventilation (VE_peak_), peak respiratory exchange ratio (RER_peak_), HR_peak_, rating of perceived exertion (RPE) and [La]_peak_ (Table 2).

**Table 2:** Variables indicating the level of exertion at exhaustion

Variable	Pre-training (mean ± SD)	Post-training (mean ± SD)	Phlebotomy (mean ± SD)
Upright cycling			
HR_peak_ (bpm)	196 ± 7	193 ± 9	193 ± 10
VE_peak_ (l min^−1^)	146 ± 40	150 ± 39#	148 ± 33
RER_peak_	1.22 ± 0.06	1.21 ± 0.05	1.19 ± 0.06
[La]_peak_ (mmol l^−1^)	12.3 ± 1.9	12.7 ± 2.1	12.8 ± 2.1
RPE	19.5 ± 0.7	19.5 ± 0.7	19.6 ± 0.5
Supine cycling			
HR_peak_ (bpm)	184 ± 9	181 ± 12	181 ± 13
VE_peak_ (l min^−1^)	118 ± 31	123 ± 29	122 ± 27
RER_peak_	1.20 ± 0.06	1.18 ± 0.05	1.15 ± 0.05*
[La]_peak_ (mmol l^−1^)	10.9 ± 2.1	11.5 ± 2.2	11.1 ± 2.8
RPE	19.5 ± 0.8	19.6 ± 0.7	19.5 ± 0.8

*N* = 11, *HR*_*peak*_ peak heart rate (10-s average), *[La]*_*peak*_ peak blood lactate concentration, *RPE* rating of perceived exertion using the Borg scale (6–20), *RER*_*peak*_ peak respiratory exchange ratio (30-s average), *VE*_*peak*_ peak ventilation (30-s average)

*Significantly different from pre-training (*P* ≤ 0.05)

^#^Trend towards being different from pre-training (0.05 < *P* ≤ 0.10)

### Submaximal exercise

**Fig. 5:**
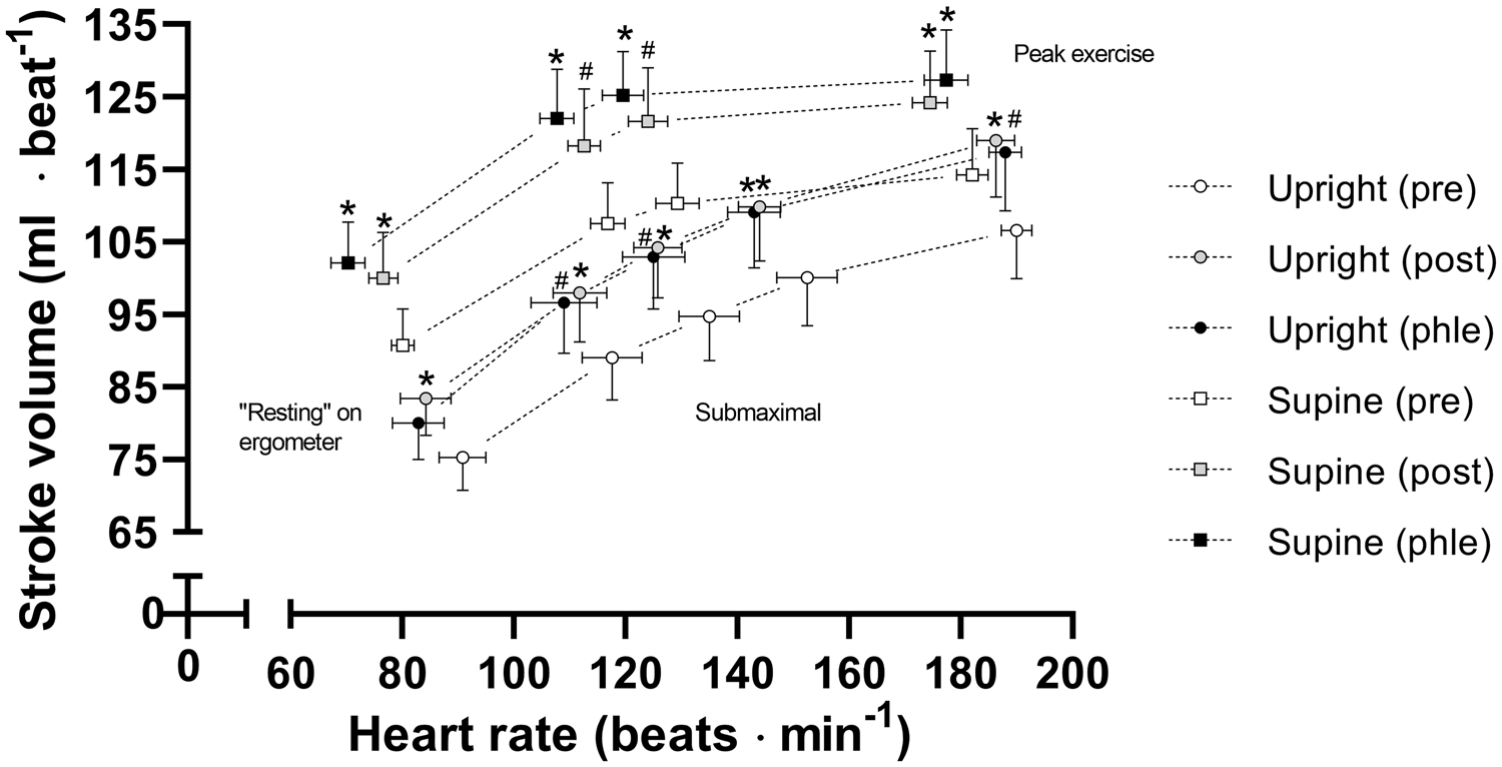
The stroke volume during upright and supine cycling as a function of heart rate during pre-training, post-training and phlebotomy exercise trials. Error bars indicate standard error of the mean. *Significant change in stroke volume from pre-training (*P* ≤ 0.05). ^#^Trend towards change in stroke volume from pre-training (0.05 < *P* ≤ 0.10). *N* = 11

During submaximal exercise, SV was increased after ET both during upright (*F*_1.6, 16.2_ = 3.7; *P* = 0.054; see Fig. 5 for post hoc tests) and supine cycling (*F*_1.4, 14.1_ = 8.2; *P* = 0.008), which was accompanied by a reduction in HR (upright: *F*_1.4, 14.4_ = 4.9; *P* = 0.032; supine: *F*_1.4, 14.0_ = 15.9, *P* < 0.001). The a-$\bar{\text{v}}$O_2_diff was unchanged during submaximal exercise (upright: *F*_1.9, 19.4_ = 1.0; *P* = 0.369; supine: *F*_1.6, 16.4_ = 0.6; *P* = 0.517).

### Cardiac morphology and function

The LV mass increased by 13 ± 17 g after ET (*P* = 0.027) and no change was observed for LV and RV chamber volumes (Table 3). Hence, an increase in LV mass-to-volume ratio (LV mass/EDV) was observed, especially after phlebotomy (*P* = 0.013), indicating concentric cardiac remodelling. All LV and RV diastolic and systolic function parameters were unchanged.

**Table 3:** Cardiac morphology and function measured at rest before and after 10 weeks of endurance training, as well as directly after phlebotomy

Variable	Pre-training (mean ± SD)	Post-training (mean ± SD)	Phlebotomy (mean ± SD)
Left ventricular morphology			
LV mass (g)	123 ± 37	137 ± 37*	
IVSd (mm)	7.4 ± 0.8	8.1 ± 0.8	
LV PWd (mm)	7.3 ± 0.9	7.7 ± 0.9	
LV EDD (mm)	49.1 ± 6.5	50.1 ± 6.2	49.9 ± 6.6
3D EDV (ml)	124 ± 35	124 ± 40	117 ± 39
3D ESV (ml)	49 ± 15	50 ± 19	46
LV mass-to-volume ratio (g ml^−1^)	1.02 ± 0.26	1.12 ± 0.20	1.19 ± 0.21*
Left ventricular systolic function			
3D ejection fraction (%)	61 ± 3	60 ± 3	61 ± 4
3D stroke volume (ml)	75 ± 21	74 ± 21	71 ± 23
Global longitudinal strain (%)	− 22.5 ± 1.4	− 22.1 ± 1.3	− 21.1 ± 1.8^#^
Left ventricular diastolic function			
E (cm s^−1^)	72.9 ± 14.5	73.2 ± 17.9	69.2 ± 13.3
A (cm s^−1^)	47.2 ± 5.4	47.3 ± 5.0	46.2 ± 10.4
E/A ratio	1.6 ± 0.4	1.6 ± 0.4	1.6 ± 0.4
E′ (cm s^−1^)	12.5 ± 2.1	12.0 ± 2.6	11.6 ± 1.9
A′ (cm s^−1^)	7.8 ± 1.1	7.5 ± 1.4	7.2 ± 1.5
E/E' ratio	5.8 ± 0.6	6.1 ± 1.0	6.1 ± 1.2
Right ventricular morphology and function			
RV end-diastolic area (cm^2^)	19.3 ± 4.3	20.5 ± 3.0	20.3 ± 4.1
RV end-systolic area (cm^2^)	11.2 ± 2.3	12.0 ± 1.8	11.5 ± 2.4
RV fractional area change (%)	42 ± 4	42 ± 3	43 ± 2
TAPSE (cm)	2.35 ± 0.18	2.31 ± 0.22	2.35 ± 0.26
Atrial morphology			
Left atrial volume (ml)	46 ± 9	50 ± 8	45 ± 8
Right atrial area (cm^2^)	15.7 ± 2.9	15.1 ± 2.4	15.5 ± 2.8

LV mass, IVSd and LV PWd are reported as the mean of the post-training and phlebotomy measurements

*N = 11* except for global longitudinal strain and RV parameters (*N* = 10), *E and A* peak mitral inflow velocity during early diastole and during atrial systole, respectively, *E′ and A′* myocardial peak velocity during early diastole and atrial systole, respectively, *EDV* end-diastolic volume, *ESV* end-systolic volume, *EDD* end-diastolic diameter, *IVSd* interventricular septum thickness in end-diastole, *LV* left ventricular, *PWd* posterior wall thickness in end-diastole, *RV* right ventricular, *TAPSE* tricuspid annular plane systolic excursion

*Significantly different from pre-training (*P* ≤ 0.05)

^#^Trend towards being different from pre-training (0.05 < *P* ≤ 0.10)

### Muscular adaptations

**Fig. 6:**
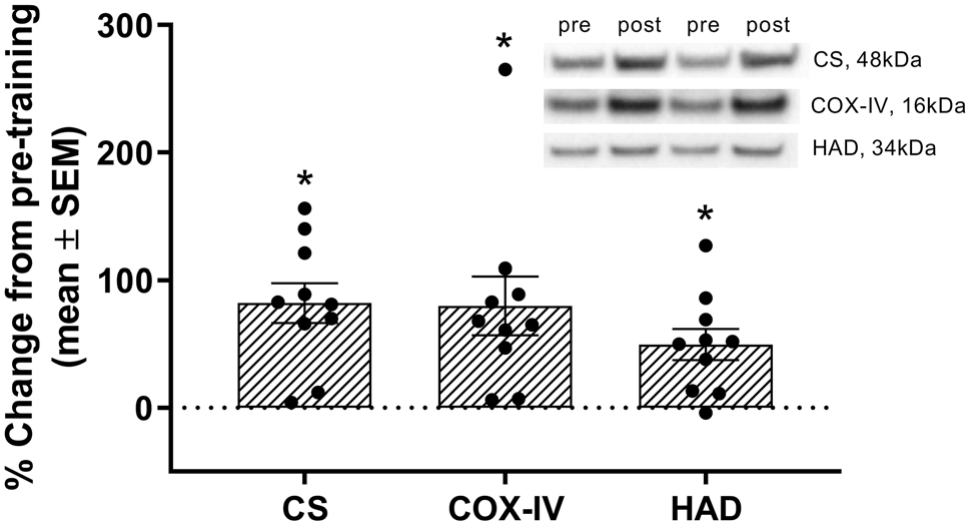
The percentage change in protein content and representative Western blots for citrate synthase (CS), cytochrome c oxidase subunit 4 (COX-IV) and hydroxyacyl-CoA dehydrogenase (HAD) from pre- to post-training. The error bars indicate standard error of the mean. Black circles indicate individual responses. *All proteins increased from pre- to post-training (*P* < 0.001). *N* = 10

ET increased the protein content of the mitochondrial enzymes CS (*P* < 0.001), COX-IV (*P* = 0.007) and HAD (*P* = 0.003; Fig. 6). Fibre cross-sectional area increased (Table 4), both in type I (*P* = 0.039) and type II (*P* = 0.023) muscle fibres. Despite increased cross-sectional area, the capillary density (capillaries mm^−2^) was maintained (*P* = 0.772), due to a trend towards increased capillary-to-fibre ratio (*P* = 0.065).

**Table 4:** Muscle morphology and capillarisation before and after 10 weeks of endurance training

Variable	Pre-training (mean ± SD)	Post-training (mean ± SD)
Fibre size (μm^2^)		
All fibres	4176 ± 818	4799 ± 1032*
Type I	3870 ± 468	4479 ± 720*
Type II	4406 ± 1218	5027 ± 1562*
Capillaries around a fibre		
All fibres	3.4 ± 0.8	3.8 ± 0.8
Type I	3.4 ± 0.7	3.8 ± 0.8
Type II	3.3 ± 0.7	3.7 ± 1.0
Capillaries mm^−2^	337 ± 86	345 ± 67
Capillary-to-fibre ratio	1.38 ± 0.31	1.62 ± 0.40#

*N* = 10

*Significantly (*P* ≤ 0.05)

^#^Trend towards (0.05 < *P* ≤ 0.10) change from pre- to post-training

## Discussion

The primary findings of the present study are (1) the ET-induced increase in $\dot{V}$O_2peak_ was preserved despite removing the ET-induced increases in Hb_mass_ and BV by phlebotomy. Therefore, improvements in $\dot{V}$O_2peak_ and $\dot{Q}$_peak_ cannot be exclusively attributed to BV expansion. (2) The improvements in $\dot{V}$O_2peak_ were mainly accounted for by the increase in $\dot{Q}$_peak_ and SV_peak_, independent of posture. (3) The ET induced concentric cardiac remodelling including an increase in LV mass, with preserved chamber dimensions measured at rest, which probably contributed to the increase in SV_peak_.

### Why was $\dot{\mathrm{V}}$O_2peak_ maintained after reducing Hb_mass_ to pre-training values?

Despite normalising Hb_mass_ and BV, the ET-induced improvements in $\dot{V}$O_2peak_ and $\dot{Q}$_peak_ were maintained during upright cycling. This contradicts studies using a similar experimental design, which suggest that $\dot{V}$O_2peak_ and $\dot{Q}$_peak_ return to pre-ET levels after removing the ET-induced elevations in BV (Bonne et al. 2014) and RBCV (Montero et al. 2015a). We speculate that the discrepancies between studies originate from the magnitude of the ET-induced BV expansion. For Bonne et al. (2014) and Montero et al. (2015a), the ET-induced increases in BV were 382 ml (7%) and 310 ml (6%), respectively, compared to 181 ml (4%) in the present study. Yet, improvements in $\dot{V}$O_2peak_ (9–11%) and $\dot{Q}$_peak_ (7–10%) during upright cycling were similar in the three studies. Thus, ET-induced increases in $\dot{V}$O_2peak_ and $\dot{Q}$_peak_ of this magnitude does not depend on BV expansion alone, and $\dot{Q}$_peak_ improved partly due to different mechanisms in the three studies. This is supported by increased LV mass in the present study, as opposed to no change in the study by Bonne et al. (2014), and suggests that multifactorial mechanisms explain the ET-induced increases in $\dot{Q}$_peak_ and $\dot{V}$O_2peak_ as suggested in classical studies (Saltin et al. 1968).

Because of the small BV withdrawal, little hypovolemia-induced impairment on venous return was likely elicited in our subjects. Supported by no reductions in $\dot{Q}$_peak_, $\dot{V}$O_2peak_ and submaximal SV during upright cycling after phlebotomy. Consequently, we were unable to test one of our main hypotheses that when $\dot{V}$O_2peak_ and $\dot{Q}$_peak_ during upright cycling were reversed to pre-ET levels after phlebotomy, the improvements would be preserved during supine cycling owing to the beneficial gravitational effects on venous return.

Apparently, it may be that the cardiovascular system can maintain venous return to the heart, despite small BV reductions by redistributing venous volumes through increased vasomotor activity acting on capacitance vessels. This is supported by the unchanged LV EDV, SV and peak mitral inflow velocity during early diastole from before to just after the phlebotomy procedure. There is, however, some uncertainty in extrapolating these responses measured during supine rest to upright peak exercise, where LV filling times are shorter and exert a major challenge on cardiac preload (Gledhill et al. 1994). Similarly, indications of maintained venous return despite small reductions in BV have been observed in studies examining fluid loss after heat stress and prolonged exercise (Saltin 1964; Saltin and Stenberg 1964). In those studies, a small-to-moderate reduction in PV was not sufficient to decrease $\dot{Q}$_peak_, assessed in normothermic conditions. Thus, it was argued that an effective contribution from the muscle pump and increased vasomotor activity enabled a normal SV despite reduced BV. In contrast to small BV losses, phlebotomy of one unit of blood (450 ml) or more reduces $\dot{V}$O_2peak_ by lowering venous return (Kanstrup and Ekblom 1984; Krip et al. 1997). Therefore, a certain threshold might exist, at which small reductions in BV are not detrimental to venous return, acting as a mechanism for the circulation to cope with small BV losses and PV reductions induced by, e.g., dehydration.

Although Hb_mass_ and BV were restored to pre-ET levels, transcapillary fluid shifts may occur during and after the phlebotomy procedure. The first exercise trial started 45 min after phlebotomy as compared with ~ 15 min in the studies by Bonne et al. (2014) and Montero et al. (2015a). To obtain an indication of fluid shifts, we measured [Hb] repeatedly during the phlebotomy day in six of the subjects. From before (15.6 ± 1.0 g dl^−1^) to just after the phlebotomy procedure, the venous [Hb] was slightly decreased (15.2 ± 1.0 g dl^−1^; *P* = 0.19), with no further change until initiation of the first (15.2 ± 1.2 g dl^−1^) and second (15.2 ± 1.1 g dl^−1^) cycling exercise trials. Therefore, at the end of, or gradually during the phlebotomy procedure, a gross movement of fluid from the interstitium to the plasma appears to have counteracted the blood withdrawal. Hence, the PV (and BV) may have been slightly higher during the phlebotomy exercise trials than reported in the present study, which may have contributed to the maintained $\dot{Q}$_peak_. However, since the reduction in [Hb] exclusively occurred from before to just after the phlebotomy, this mechanism has likely also affected the subjects in the Bonne et al. (2014) and Montero et al. (2015a) studies. Also, the Hb_mass_ was re-established to pre-ET levels independent of potential PV shifts.

We have focused on the role of BV for $\dot{Q}$_peak_, i.e. one of the main determinants of venous return and cardiac preload. However, an ET-induced reduction in afterload through a lowering of total peripheral resistance may also increase $\dot{Q}$_peak_. For instance, after ET of both legs separately, Klausen et al. (1982) found a reduction in mean arterial pressure (MAP) and total peripheral resistance that likely contributed to the increased $\dot{Q}$_peak_ after ET. This mechanism can also have facilitated the increased $\dot{Q}$_peak_ in the present study.

### The magnitude of haematological adaptations

The changes in BV reported after 6–8 weeks ET (3–4 sessions week^−1^) typically range from 140 to 550 ml (Bonne et al. 2014; Helgerud et al. 2007; Montero et al. 2017, 2015a; Montero and Lundby 2017b), whereas a meta-analysis reported a mean increase of 267 ml after ~ 15 weeks of ET (range 1–51 weeks) (Montero and Lundby 2017a). A complex interplay of mechanisms causes the BV expansion in ET that may be affected by the training program, the training status of the subjects and their nutritional status (Montero and Lundby 2018; Sawka et al. 2000). Although our BV expansion was within the expected range, the smaller increase compared to that by Bonne et al. (2014) and Montero et al. (2015a) is unlikely caused by iron deficiency, as indicated by the normal and maintained ferritin levels. Lean body mass was maintained, indicating sufficient protein and caloric intake during the ET period. Over 10 weeks, the subjects performed 27–30 ET sessions as compared to only 18–20 sessions over 6 weeks in the studies by Bonne et al. (2014) and Montero et al. (2015a), using a similar training intensity. Hence, the training intensity and the total volume and length of the training intervention cannot explain the different findings.

### Central vs peripheral limitations to $\dot{\mathrm{V}}$O_2peak_

There were no statistically significant changes in estimated a-$\bar{\text{v}}$O_2_diff from pre- to post-ET, potentially indicating no substantial contribution of peripheral adaptations to the improvements in $\dot{V}$O_2peak_. Based on the average of all maximal exercise tests conducted pre- (*n* = 2; upright and supine cycling) and post-ET (*n* = 4; before and after phlebotomy), $\dot{V}$O_2peak_ increased by 318 ± 147 ml min^−1^. Of this increase, the increase in $\dot{Q}$_peak_ account for 221 ± 202 ml min^−1^. Hence, on average, a-$\bar{\text{v}}$O_2_diff was elevated by 5 ± 12 ml l^−1^ (154 ± 22 vs 159 ± 25 ml l^−1^) and account for 30% of the increase in $\dot{V}$O_2peak_. Therefore, our data support that $\dot{V}$O_2peak_ is mainly limited by convective O_2_ delivery (Montero et al. 2015b; Mortensen et al. 2005), but also supports calculations indicating that 70–75% of the limitations lie within the central circulation and that 25–30% are determined by peripheral factors (di Prampero 2003; di Prampero and Ferretti 1990).

O_2_ extraction and blood flow are interdependent. For example, by decreasing $\dot{\mathrm{Q}}$_peak_ and leg blood flow using β-adrenergic blockade, systemic and leg a-$\bar{\text{v}}$O_2_diff increases during submaximal and maximal exercise, facilitated by increased erythrocyte capillary mean transit time (MTT) (Ekblom et al. 1972; Pawelczyk et al. 1985). In the present study, muscle fibre hypertrophy was accompanied by only a minor increase in the capillary-to-fibre ratio, causing no change in capillary density. If we calculate the capillary volume within the leg muscle mass engaged during cycling (Boushel et al. 2014) and subtract a non-leg blood flow of 6.5 l min^−1^ from the total $\dot{Q}$_peak_ (Calbet et al. 1985,2006; Lundby et al. 1985; Mortensen et al. 2005), there would be a trend towards shorter erythrocyte MTT after ET during upright peak exercise (508 ± 138 vs 452 ± 132 ms before and after ET, respectively). Therefore, due to reduced time for O_2_ unloading, peripheral adaptations such as increased muscle oxidative capacity (CS and COX-IV) may have been crucial in maintaining the pre-ET level of a-$\bar{\text{v}}$O_2_diff. This is substantiated by a correlation between the percent change in a-$\bar{\text{v}}$O_2_diff during upright cycling and the percent change in CS content from before to after ET (*r* = 0.73; *n* = 10; *P* = 0.017). Further improvements in a-$\bar{\text{v}}$O_2_diff, at least large enough to evoke statistical significance, may likely only be detected if peripheral adaptations largely surpass the changes in $\dot{Q}$_peak_ and peripheral blood flow.

After years of training, elite endurance athletes have a higher leg O_2_ extraction than untrained individuals (> 90% vs ~ 70%, respectively) (Calbet et al. 2005; Roca et al. 1985). A similar situation can be evoked by relative short periods of one-legged ET inducing robust peripheral adaptations without stimulating the central circulation, and improve leg a-$\bar{\text{v}}$O_2_diff by 5–10 ml l^−1^ (Klausen et al. 1982; Rud et al. 2012). Thus, ET improves the muscles’ ability to extract O_2_ but may be masked by improvements in $\dot{Q}$_peak_ and peripheral blood flow after short periods of whole-body ET (Montero et al. 2015b). Furthermore, the improvements seen after 7–8 weeks of one-legged ET by Rud et al. (2012) and Klausen et al. (1982) were in the range of 5–10 ml l^−1^ as assessed by arterial and femoral venous blood sampling. Accordingly, small potential improvements in systemic a-$\bar{\text{v}}$O_2_diff, as indicated in the present study (5 ml l^−1^), may be difficult to detect when calculated from $\dot{V}$O_2peak_ and non-invasively determined $\dot{Q}$_peak_.

### Cardiac remodelling

LV mass increased without any change in EDV. This contradicts the classic model of athletic cardiac remodelling that predicts increased EDV following ET due to the haemodynamic stimulus of volume loading on the ventricles (Morganroth et al. 1975). However, longitudinal studies demonstrate concentric remodelling at the commencement of ET, with the adaptations gradually switching into eccentric remodelling. For instance, after 3 months of ET, Arbab-Zadeh et al. (2014) found a 10% increase in LV mass-to-volume ratio before it returned to pre-ET levels after 9–12 months of ET. Therefore, with an increase in LV mass-to-volume ratio of 14% after 10 weeks of ET, our data support that the initial ET-induced cardiac remodelling is concentric (Arbab-Zadeh et al. 2014; Bjerring et al. 2019; Weiner et al. 2015).

Despite unchanged LV EDV and diastolic and systolic functional parameters at rest, the submaximal exercise SVs and SV_peak_ were increased after ET. This indicates an increased capacity of the heart to utilise the Frank–Starling mechanism to increase SV from rest to peak exercise after ET (Crawford et al. 1985; Rerych et al. 1980). This could be due to increased chamber compliance (Arbab-Zadeh et al. 2014; Levine et al. 1991), increased filling rates (Ferguson et al. 2001; Gledhill et al. 1994), a lower rise in MAP during exercise due to reduced total peripheral resistance (Klausen et al. 1982) or a combination. Since the SV_peak_ was elevated even after phlebotomy, increased filling rates through expanded BV are unlikely to have made any major contribution. However, with the present experimental design, we cannot determine whether increased LV mass, enhanced venous return through other mechanisms than elevated BV, reduced total peripheral resistance, or improved qualitative properties of the heart (e.g. contractility, compliance, faster ventricular relaxation) were facilitating the elevated SV_peak_ and $\dot{Q}$_peak_.

### Study considerations

Although Hb_mass_ and BV were restored to pre-ET levels by phlebotomy, transcapillary fluid shifts may have occurred, and it is uncertain whether BV normalisation was preserved during exercise. Some of the subjects were unfamiliar with cycling exercise before the study, and no one had tried supine cycling. Therefore, we cannot exclude the possibility that some subjects became better able to maintain venous return and cardiac filling at peak exercise after ET due to familiarisation. The findings were obtained from a small sample size. But the main finding was that the ET-induced increase in $\dot{V}$O_2peak_ was preserved despite removing the increase in Hb_mass_, and this conclusion is based on methods with low typical error ($\dot{V}$O_2peak_ 2.9% and Hb_mass_ 1.1%). Impedance cardiography is associated with a larger measurement error than the invasive gold-standard methods (Del Torto et al. 2019; Richard et al. 2001). Besides, when calculating the a-$\bar{\text{v}}$O_2_diff from $\dot{V}$O_2_ and $\dot{Q}$ inherent of its measurements errors, a larger measurement error is expected as compared with deriving a-$\bar{\text{v}}$O_2_diff using arterial and venous blood sampling.

## Conclusion

Improvements in $\dot{V}$O_2peak_ and $\dot{Q}$_peak_ following short-term ET do not depend on Hb_mass_ and BV expansions in untrained individuals. $\dot{V}$O_2peak_ increased primarily through increased $\dot{Q}$_peak_ but also through a widened a-$\bar{\text{v}}$O_2_diff, explaining ~ 70% and ~ 30% of the improvements, respectively, and were potentially mediated by cardiac remodelling and mitochondrial biogenesis.

## Abbreviations

a-$\bar{\text{v}}$O_2_diff: Arteriovenous oxygen difference
BV: Blood volume
CO: Carbon monoxide
COX-IV: Cytochrome c oxidase subunit 4
CS: Citrate synthase
EDV: End-diastolic volume
ESV: End-systolic volume
ET: Endurance training
HAD: Hydroxyacyl-CoA dehydrogenase
[Hb]: Haemoglobin concentration
Hb_mass_: Haemoglobin mass
HR: Heart rate
HR_peak_: Peak heart rate
[La]: Blood lactate concentration
LV: Left ventricle
MCHC: Mean corpuscular haemoglobin concentration
MTT: Mean transit time
PV: Plasma volume
$$\dot{Q}$$: Cardiac output
$\dot{Q}$_peak_: Peak cardiac output
RBCV: Red blood cell volume
RER_peak_: Peak respiratory exchange ratio
RPE: Rating of perceived exertion
RV: Right ventricle
SD: Standard deviation
SV: Stroke volume
VE_peak_: Peak ventilation
$$\dot{V}$$O_2_: Oxygen uptake
$\dot{V}$O_2__peak_: Peak oxygen uptake
$\dot{W}$_peak_: Peak power output
